# Peripheral immune tolerance by prolactin-induced protein originated from human invariant natural killer T cells

**DOI:** 10.1080/21655979.2021.1875664

**Published:** 2021-01-28

**Authors:** Hyeong-Woo Lee, Juhyun Shin, Brian S. Wilson, Jae-Wook Oh

**Affiliations:** a Department of Animal Science and Technology, Konkuk University, Seoul, Republic of Korea; b Speegenebio, Co., Ltd, Seongnam, Republic of Korea; c Department of Pathology, Immunology, and Laboratory Medicine, University of Florida, Gainesville, FL, USA; d Department of Stem Cell and Regenerative Biotechnology and Animal Resources Research Center, Konkuk University, Seoul, Republic of Korea

**Keywords:** Natural killer T cell, regulatory T cell, immune tolerance, dendritic cell, prolactin-induced protein

## Abstract

invariant natural killer T (iNKT) cells have been reported to regulate a diverse set of immunological responses. iNKT cell dysfunction in cytokine secretion is linked to the development of autoimmunity, an immune response against its own tissue. Interestingly, CD4^+^ iNKT cells preferentially secrete regulatory cytokines. Here we investigated what kind of secreting factors of it are involved in dendritic cell (DC) maturation to regulate immune responses. We found one of them, prolactin induced protein (PIP), from the supernatants of cultured CD4^+^ iNKT cells. It was validated using RT-quantitative real-time polymerase chain reaction (RT-qPCR) and western blot analysis. Subsequent analysis upon PIP treatment was performed using fluorescence-activated cell sorting (FACS) analysis. We identified PIP as one of strong candidates for inducing DC maturation, to similar level to lipopolysaccharide, an already known candidate molecule. Recombinant PIP recapitulated natural function, and induction of DC differentiation by both recombinant and purified PIP was blocked by anti-Toll-like receptor (TLR)2 antibody (Ab), but not by anti-TLR4/5 or anti-receptor Ab for advanced glycation end product Ab. Interestingly, PIP induced the differentiation of naïve T cells into CD4^+^ CD25^+^ Foxp3^+^ regulatory T cells and reduced the number of helper T (Th)1 and Th17 cells produced by Pam3CysSerLys4. Take in together, these results suggest that PIP is an important factor that mediates immunoregulation by iNKT cells through TLR2-mediated signaling.

## Graphical abstract

**Figure:**
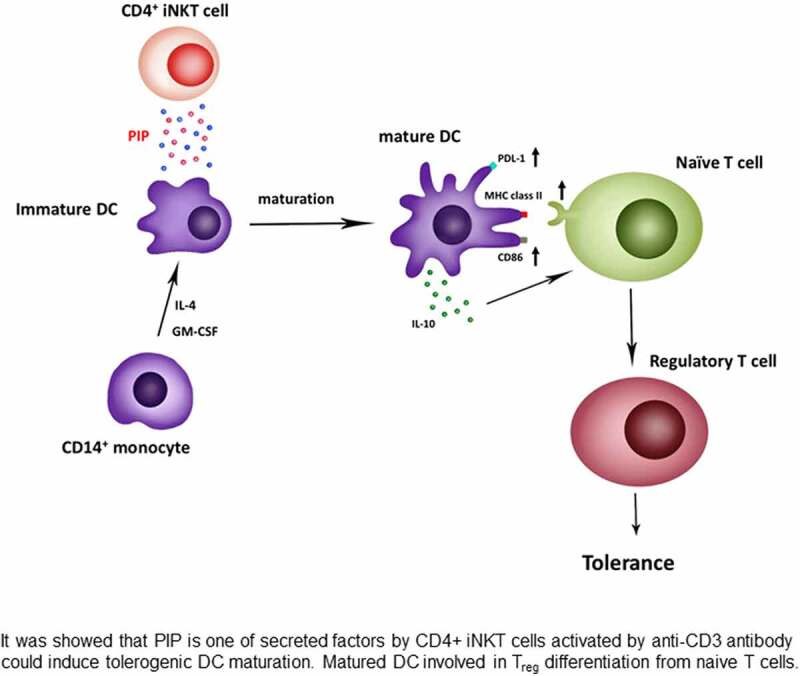

## Introduction

Human invariant natural killer T (iNKT) cells are a subset of T cells that characteristically express invariant Vα24-Jα15 T-cell receptor (TCR). In addition to several cell surface proteins usually found on NK cells [1] and share phenotypic and functional homology with both T and NK cells. iNKT cells are restricted by the non-polymorphic class Ib molecule cluster of differentiation (CD)1d [2–4] and recognize glycolipid antigens presented by CD1d [4], resulting in the rapid production of numerous cytokines, including interleukin (IL)-4 and interferon (IFN)-γ [5]. This rapid secretion of cytokines implies that iNKT cells may play an important role in modulating subsequent immune responses and related immunopathologies. iNKT cells are regarded as immuneregulators in tumor immunity [6], diabetes [7–9], and protection against bacteria (e.g., *Mycobacterium tuberculosis*) [10], viruses (e.g., *Hepatitis B* and HIV) [11], and parasitic infections (e.g., *Malaria*) [12].

While iNKT cells have been shown to significantly contribute to peripheral tolerance, their regulatory effects are not precisely understood. Soluble factors secreted by human iNKT cells lead to the differentiation of human peripheral blood monocytes into myeloid antigen-presenting cells (APCs) that have suppressive properties [13]. Additionally, human iNKT cells control primary human peripheral blood monocytes to differentiate into subpopulations of cells such as immature myeloid dendritic cells (mDCs) [14]. iNKT cell activation following the recognition of CD1d expressed on monocytes has been shown to result in the secretion of IL-13 and granulocyte macrophage-colony stimulating factor (GM-CSF), which triggers monocyte differentiation. The exposure of iNKT cells to lipopolysaccharide (LPS) led to changes in immature mDCs typically associated with DC maturation, including the upregulation of C-C chemokine receptor type 7 and CD83 [15] and transport of MHC class II molecules to the cell surface [13]. Importantly, a separate study showed failures in standard DC maturation in both humans and rodents with autoimmune diabetes. Furthermore, the addition of highly mature DCs protected non-obese diabetic (NOD) mice from disease. Hence, defects in CD1d-restricted T cells and APCs may trigger the progression of pathogenic type 1 diabetes and autoimmune T cells [8].

Prolactin-induced protein (PIP) is a 17-kDa glycoprotein present in various human body fluids since it produced in apocrine glands [16]. This protein is also known as gross cystic disease fluid protein 15, secretory actin-binding protein, and gp17, indicating its functional roles in human reproductive and immunological systems [17–19]. PIP binds to many proteins such as actin [20], myosin, keratin, tropomyosin, immunoglobulin (Ig) G [21], and human zinc-alpha2 glycoprotein (ZAG) [22]. The ability of PIP to bind to IgG-Fc suggests that it may have an immunomodulatory role. Moreover, PIP binds to CD4 and reported to block CD4-mediated programmed T cell death; thus, it plays a functional role in the immune response [23]. PIP expression has been well studied in breast and prostate cancers, and it is considered a marker for both. The mitogenic activity of PIP in breast cancer cell lines suggests that it actively contributes to tumor proliferation [24]. Low expression of PIP in human seminal plasma is suggested to be associated with infertility [25]. Further, formation of the ZAG–PIP complex in the seminal plasma indicates its possible role in sperm motility [23]. Consequently, PIP has been suggested to play a role in various biological processes, including reproduction, immunoregulation, antimicrobial activity, apoptosis, and tumor progression; however, its exact physiological function remains still unclear. The relative expression levels of the PIP gene were found to be highest in salivary glands (56.42%), followed by the levels in the lacrimal gland (16.2%), prostate gland (8.63%), muscle (2.87%), trachea (2.85%), mammary gland (1.84%), lung (0.51%), and other organs (10.67%) [17].

In this study, we aimed to identify the factors produced by CD4^+^ iNKT cells that could induce tolerogenic DCs that allowed regulatory T cells to be generated. Especially, characterized the role of PIP in the immunoregulation of CD4^+^ iNKT cells and immune tolerance. Before initiating this study, we also investigated the role of chemokines and cytokines secreted by CD4^+^ iNKT cells in DC maturation.

## Materials and methods

### Cell lines

The CD1d-restricted T cell clones were generated by the cell sorting method using MoFlo (Becton Dickinson, Mountainview, CA, USA). Briefly, NKT cells were sorted using 6B11fluorochrome-conjugated monoclonal Abs that were specific for the invariant complementarity determining region (CDR)3 region of Vα24JαQ [25]. The sorted single cells were cultured with a mixture of irradiated (5,000 rads) allogeneic peripheral blood mononuclear cells (PBMCs) at a density of 75,000 cells per well. The NKT clones were stored in liquid nitrogen until further use. When thawed, the clones were pulsed using 100 ng/ml α-galactosylceramide (α-GalCer; KRN7000, Avanti Polar Lipids, Alabaster, AL, USA) and gamma-irradiated PBMCs. This study was approved by the Committees on Ethics of Konkuk University. This study was conducted according to the principles expressed in the Declaration of Helsinki. The study procedures, potential risks, and benefits were explained to all participants. Furthermore, all data were analyzed anonymously; no volunteer was identified by name.

### Culture of NKT cell clones and transfection

NKT cell clones were expanded by culturing in RPMI 1640 medium (BioWhittaker, Walkersville, MD, USA) supplemented with 10% (v/v) heat-inactivated fetal bovine serum (FBS; Atlanta Biologicals, Norcross, GA, USA), 2.5 × 10^7^ irradiated allogeneic PBMCs, 2 mM L-glutamine, 100 ng/ml α-GalCer, 10 mM HEPES buffer, 100 μg/ml streptomycin sulfate, and 100 U/ml penicillin [26]. The PBMCs used as feeder cells for NKT cells were isolated by Ficoll-Paque (Amersham Pharmacia Biotech, Uppsala, Sweden) density gradient centrifugation from venous blood collected from healthy donors. Cells were incubated in a humidified chamber with 5% CO_2_ at 37°C. After 18‒24 h incubation, 10 U/ml human IL-7 (Roche, Mannheim, Germany), and 50 U/ml human recombinant IL-2 (Roche) were added to the feeder cells and co-cultured NKT cells. On day 5, nearly half of the medium was substituted with fresh medium supplemented with 10 U/ml IL-7 and 50 U/ml IL-2. On days 10‒14, NKT cells were split for the subsequent expansion. Cell purity was assessed by flow cytometry using CD4^−^, CD8^−^, and 6B11fluorochrome-conjugated antibodies (Abs). Cell transfection with siRNA (S100 calcium-binding protein A8, PIP Trilencer-27 Human siRNA, and S100A8; OriGene, Rockville, MD, USA) against S100A8 and PIP was performed following the manufacturer’s instructions, and cells were allowed to recover for 24 h before use.

### Reverse transcription-polymerase chain reaction (RT-PCR) analysis

Total RNA was isolated with an RNeasy Mini Kit (Qiagen Co., Valencia, CA, USA) following the manufacturer’s protocol. For RT-PCR, 1 μg of total RNA, PIP-Forward (sense, GCT CAG GAC AAC ACT CGG AA) and PIP-Reverse (antisense, ATA ACA TCA ACG ACG GCT GC), and reverse transcriptase SuperScript II (Invitrogen, Carlsbad, CA, USA) were added, and 34 cycles of initial denaturation at 94°C for 30s, annealing at 55°C for 30s, and extension at 72°C for 60s were run to amplify the *PIP* gene.

### Separation of anti-CD3-activated NKT cell culture supernatant

To prepare the supernatant from CD4^+^ iNKT cells activated with anti-CD3 Ab, cultures were treated with 100 ng/ml of anti-CD3 Ab (Ancell, Bayport, MN, USA) and incubated overnight at 4°C. After washing the plates three times with RPMI 1640 medium containing 10% (v/v) FBS, CD4^+^ iNKT cells were added and cultured at 37°C and 5% CO_2_. After incubation for 7 days, the supernatant was collected and filtered using a strile 0.2 μm filter membrane (Thomas Scientific, Swedesboro, NJ, USA) to eliminate the cell debris.

### Isolation of DC maturation factors by fast protein liquid chromatography (FPLC)

To isolate the DC maturation factors, protein samples were applied to an AKTA Explorer 100 FPLC system (GE Healthcare, Piscataway, NJ, USA). Briefly, 30 ml anti-CD3 Ab-activated supernatant from NKT cells was applied to a HiTrap diethylaminoethanol fast flow (DEAE FF) column (5 ml, GE Healthcare), and the DC maturation factors were eluted with 20 mM Tris-Cl (pH 8.0) containing 1 M NaCl. From each collection tube, 50 μl of supernatant was used to test the allogeneic immature DC maturation effect. Samples that showed a DC maturation effect were concentrated by dialysis with 20 mM Tris-Cl buffer at 4°C overnight after freeze-drying. The active region was again loaded to a HiTrap CM FF column (5 ml, GE Healthcare) and corrected by DEAE anion column chromatography. Active regions that were prepared for cation chromatography [27] were reloaded onto a HiTrap Blue HP (1 ml, GE Healthcare) and finally eluted with 1.5 M KCl (pH 7.0) containing 50 mM KH_2_PO_4_. The protein contents of the final active regions were analyzed by mass spectrometry (MS).

### MS analysis

Partially purified DC maturation factors from the supernatant of CD4^+^ iNKT cells were loaded onto a 12% sodium dodecyl sulfate-polyacrylamide gel electrophoresis (SDS-PAGE) gel and run at 35 mA for 40 min. Following electrophoresis, gels were stained with Coomassie Brilliant Blue G-250 (Boston BioProducts, Worcester, MA, USA) before the areas of interest were excised and digested ‘in-gel’ with trypsin, and the resulting peptides were spotted onto matrix-assisted laser desorption/ionization (MALDI) target plates [28]. An A4700 Proteomics Analyzer with TOF-TOF Optics (Applied Biosystems, Framingham, MA, USA) was used to obtain MALDI-MS/MS spectra of tryptic peptides in reflector positive mode, with the 10 most intense peaks in the MS spectrum automatically selected as singly charged, because this is the dominant species produced by the analyzer. MS/MS data from each in-gel digest were processed by GPS Explorer v3.0 software (Applied Biosystems) to identify peaks representing proteins of 20 Da to 60 kDa below the precursor mass with a minimum S/N of 20. A protein was considered positively identified if the GPS Explorer total ion score (generated by GPS Explorer) confidence interval exceeded 99.9% and if at least one peptide had a MASCOT expectation value of less than 0.05, indicating that the probability of a random match was much less than 0.05. Protein identities were confirmed by searching for matches in the National Center for Biotechnology Information (NCBI) database.

### Generation of DCs from buffy coats and maturation testing

Buffy coats were obtained from healthy donors following the institutional guidelines. PBMCs were separated by density centrifugation using a Ficoll-Paque gradient method (Amersham Pharmacia). PBMCs (7 × 10^7^) were added to 10 cm tissue culture plates in 10 ml of RPMI 1640 culture medium containing 10% heat-inactivated FBS, 100 U/ml penicillin, 2 mM l-glutamine, and 10 µg/ml streptomycin, and these cells were further incubated at 37°C with 5% CO_2_ for 1 h. The non-adherent cells were discarded, and the adherent cells were carefully collected using cell scraper. In some conditions, microbead-conjugated with anti-CD14 Ab (Miltenyi Biotech, Auburn, CA, USA) were added to the PBMCs and incubated on ice for 30 min. After washing with binding buffer [2 mM EDTA, 2% FBS, and 20 mM phosphate-buffered saline (PBS), pH 7.4], the CD14^+^ cells were isolated using an autoMACS Pro Separator (Miltenyi Biotech). Purified cells were then cultured in RPMI 1640 medium supplemented with 10% heat-inactivated FBS, 2 mM L-glutamine, 10 mM HEPES, 10 μg/ml streptomycin, 100 U/ml penicillin, 50 µM β-mercaptoethanol (ME), 5 ng/ml GM-CSF, and 10 ng/ml IL-4. Approximately half of the medium was replaced with fresh medium on days 3 and 6. To examine the DC maturation effect, non-adherent cells (immature DCs) were harvested (on day 7) and stimulated with purified supernatant from NKT cells or TNF-α. The maturation of DCs was determined by the detection of surface molecules CD86, programmed death-ligand (PDL)-1, and human leukocyte antigen (HLA)-DR by fluorescence-activated cell sorting (FACS) analysis using a FACSCalibur instrument (BD Biosciences, San Jose, CA, USA).

### Flow cytometric analysis

NKT cell clones were phenotypically characterized by staining the cell surface markers using Abs conjugated to phycoerythrin (PE) or fluorescein isothiocyanate (FITC). Briefly, cells were washed in staining buffer [PBS (pH 7.2) with 2% FBS + 0.1% sodium azide] prior to staining with the appropriate conjugated antibodies by incubating for 30 min at 4°C. Subsequently, the cells were washed twice with staining buffer [PBS (pH 7.2) with 2% FBS + 0.1% sodium azide]. FITC-conjugated Abs comprised those against IgG, CD4, and CD8, while PE-conjugated Abs consisted of those against NKT (6B11), CD3, CD4, and CD8. For DCs, HLA-DR, CD86, CD14, and PDL-1 (BD Biosciences) were used to determine maturation. Intracellular staining was performed following the manufacturer’s instructions (BD Biosciences). Isotype-matched control Abs were included for all experiments to control nonspecific binding. Dead cells were gated by forward and side scatter. Flow cytometry was performed using a FACSCalibur instrument (BD Biosciences), and data were analyzed using FlowJo software (TreeStar, Ashland, OR).

### Western blot analysis

Anti-CD3 Ab-activated CD4^+^ iNKT cells were harvested and washed with cold PBS. The cell pellet was resuspended in radioimmunoprecipitation assay buffer [50 mM Tris-HCl (pH 7.4), 1% NP-40, 150 mM NaCl, 0.1% SDS, 0.5% sodium deoxycholate, 1 mM ethylene glycol-bis(β-aminoethyl ether)-N,N,Nʹ,Nʹ-tetraacetic acid (EGTA) and 5 mM EDTA] and placed on ice for 30 min. Subsequently lysates were centrifuged at 14,000 *g* for 10 min, following which the supernatants were collected. An equal volume of Laemmli’s sample buffer (20% glycerol, 10% β-ME, 4% SDS, and 4 mg/100 ml bromophenol blue) was added to the supernatant, propr to denaturing at 95°C for 5 min. The samples were then run on a 12% SDS-PAGE gel for 40 min (35 mA). The supernatants prepared from anti-CD3 Ab-activated CD4^+^ iNKT cells were filtered through a 0.2 μm membrane, mixed with the sample buffer, and then loaded onto the SDS-PAGE gel. The proteins were electrotransferred to polyvinylidene difluoride membranes using a transfer buffer containing 25 mM Tris, 190 mM glycine, and 20% methanol at 300 mA for 2 h. After 2 h of saturation in PBS containing 5% nonfat milk and 0.05% Tween 20, membranes were incubated with anti-PIP and 1:500 (v/v) anti-beta tubulin Ab (Cell Signaling Technology, Danvers, MA) overnight at 4°C with gentle shaking. Membranes were washed three times with PBS comprising 0.05% Tween 20 and incubated with 1:1000 (v/v) horseradish peroxidase-conjugated anti-mouse IgG and 1:1000 (v/v) anti-rabbit IgG Ab (Cell Signaling) for 1 h at 20‒24°C. Proteins were spotted using the Western Lightning® Plus-ECL, Enhanced Chemiluminescence Substrate kit (PerkinElmer, Waltham, MA).

### Cytokine assay

The amounts of granulocyte (G)-CSF, GM-CSF, IL-1β, IL-2, IL-4, IL-5, IL-6, IL-7, IL-8, IL-10, IL-12, IL-17, IFN-γ, monocyte chemoattractant protein-1 (MCP-1), TNF-α, and macrophage inflammatory protein (MIP)-1β in supernatants of cultured cells were determined using a Human Cytokine 17-plex kit (Bio-Rad, Hercules, CA, USA) following the manufacturer’s instructions.

### Statistical analysis

All values are expressed as means ± standard deviations. Differences between the effects of PIP and LPS of DC maturation, Toll-like receptor (TLR) blocking, and anti- receptor for advanced glycation end products (RAGE) Ab blocking were compared using one-way ANOVA followed by Bonferroni’s multiple comparison test. A *p*-value of < 0.05 was considered statistically significant. The statistical significance of differences in T_reg_ generation was determined by *t*-test with a *p*-value cutoff of 0.05.

## Results and discussion

### CD4^+^ iNKT cells efficiently induce DC maturation

DCs are key essential mediators of the adaptive immune system of the body. In the non-activated state, such as in the absence of infection, DCs in peripheral tissues are found in a resting immature state, with partial activity to stimulate naïve T cells. However, following infection, DCs undergo a functional maturation process, which includes phenotypic changes, resulting in an enhanced potential to promote T cell responses. In the absence of inflammation or infection, there is a steady flux of immature DCs that capture and process endogenous antigens [29]. These DCs then define immunological ‘self’ and induce tolerance by specifically silencing or negatively selecting autoreactive T cells and triggering the development of T_reg_ cells [30]. Although immature DCs seem to be able to induce tolerance to foreign and self-antigens, several recent *in vivo* and *in vitro* studies have reported differentiated DCs with potent tolerogenic abilities [13,31]. Importantly, with the help of *in vivo* studies, the Bluestone group recently showed that T_reg_ cells directly interact with islet antigens-bearing DCs and this interaction preceded the inhibition of T helper (Th) cell stimulation by DCs [32]. The clarification for these apparently contradictory functions seems to lie in the remarkable ability of immature DCs to differentiate into particular functional subtypes [33]. The exchange of information between T cells and DCs is not unidirectional. While DCs are required for the adequate priming of antigen-specific lymphocyte responses, T cells are required for the optimal maturation of DCs [34]. Several reports, including the finding of the current study, have highlighted the importance of cross-talk between iNKT cells and DCs in regulating immune responses and regulating the relative percentages of DC subsets [13,35,36]. Recently, autoreactive iNKT cells were shown to directly and potently induce DC maturation.

**Figure 1.**
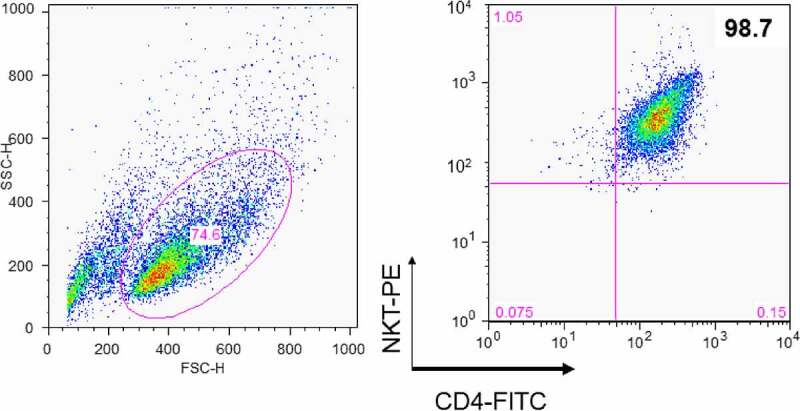
Construction of the CD4^+^ iNKT cell line. CD1d-restricted T cell clones were generated from PBMCs by cell sorting using MoFlo. Briefly, iNKT cells were sorted using 6B11 fluorochrome-conjugated Ab and sorted single cells were cultured with a mixture of irradiated (7,500 rad) allogeneic 75,000 PBMCs per well, and the clones were expanded using anti-CD3 and irradiated PBMCs. CD4^+^ iNKT cell clones were characterized phenotypically using a panel of cell surface Abs, including CD4 conjugated with fluorescein isothiocyanate (FITC) and NKT with phycoerythrin (PE)

**Figure 2.**
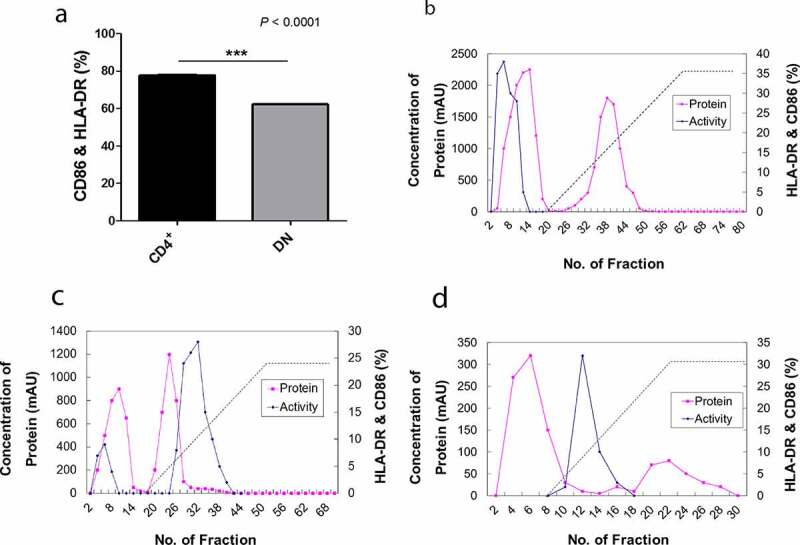
Purification of DC maturation factors secreted by CD4^+^ iNKT cells using fast protein liquid chromatography (FPLC). (a). The dendritic cell maturation effect by CD4^+^ iNKT cells and DN iNKT cells (n = 3). (b). HiTrap DEAE FF chromatography. (c). HiTrap CM FF chromatography. (d). HiTrap Blue HP chromatography. Protein contents of the active region of HiTrap CM FF were analyzed with mass spectrometry. ■ represents the protein concentration, ♦ represents the activity of maturate immature DCs, as assessed by FACS analysis using HLA-DR and CD86 monoclonal Abs

To assess the DC-maturing abilities of iNKT cells, the cells were isolated from PBMCs of healthy donors using 6B11 Ab, which reacts specifically with TCR CDR3 of human iNKT cells [25]. Subsequently, iNKT cells were separated into double negative (DN) and CD4^+^ cells using CD4 microbeads as assessed by flow cytometry (Figure 1). The soluble factors from iNKT cells showed a significant effect on DC maturation (Figure 2(a)) [8,13,14]. Consequently, we measured the representative mature DC surface markers, human CD86, and HLA-DR, using flow cytometry [13]. The expression levels of these markers were significantly upregulated following the addition of CD4^+^ iNKT cell supernatant compared to the changes observed after the addition of DN iNKT cell supernatant (Figure 2(a)). Based on these observations, we further focused on the soluble factors derived from the CD4^+^ iNKT cells.

### DC maturation factors from human CD4^+^ iNKT cells

To purify the DC maturation factors from the culture supernatant of CD4^+^ iNKT cells, supernatants were fractionated sequentially with cation, anion, and affinity chromatography using FPLC (Figure 2(b–d)), and the expression of human HLA-DR and CD86 on the surfaces of allogeneic DCs was analyzed by FACS. We analyzed the last active regions by final blue chromatography coupled to MS. Several interesting proteins were found in the chromatography fraction that showed a considerable effect on DC maturation in nine different purification experiments using allogeneic DCs. The candidate proteins were arranged according to the number of hits from these nine independent purification experiments. S100A8 was identified eight times, while PIP was detected seven times. The following proteins were detected six times: adenosine triphosphate (ATP)-binding cassette, haptoglobin, desmoplakin isoform 1, transferrin, serpin peptidase inhibitor clade A (member 3), and transthyretin. Ceruloplasmin, mesotrypsin, hemopexin, orosomucoid 1, and serine (or cysteine) proteinase (clade B) were each detected five times (Supplementary Table 1). Overall, we characterized the function of S100A8 in T_reg_ generation [35]. Since PIP has been reported to bind to CD4, block CD4-mediated programmed T cell death, and play a role in immune responses, we were interested in the ability of this protein to influence DC maturation [23].

### PIP demonstrates an effect on DC maturation

**Figure 3.**
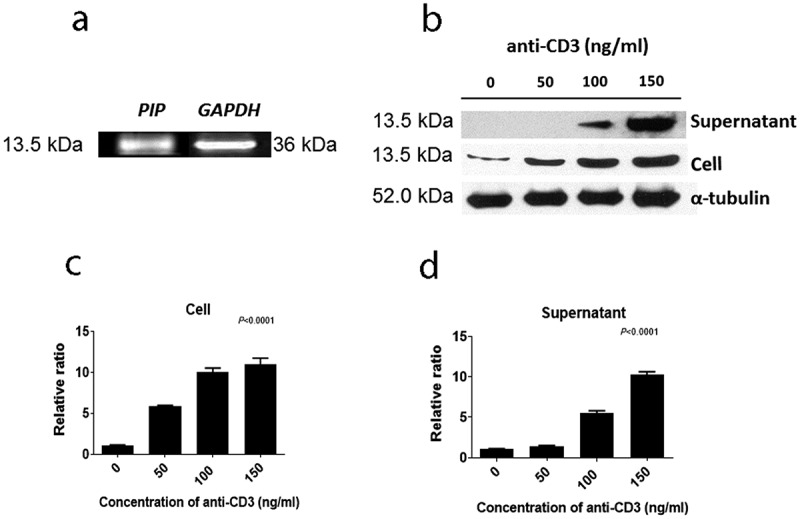
Expression of prolactin-induced protein (PIP) in CD4^+^ iNKT cells and their culture supernatant. (a). RT-PCR, (b). Western blot, (c). relative expression of PIP and (d). secreted levels of PIP, according to the concentration of anti-CD3 Ab. GAPDH: glyceraldehyde 3-phosphate dehydrogenase

**Figure 4.**
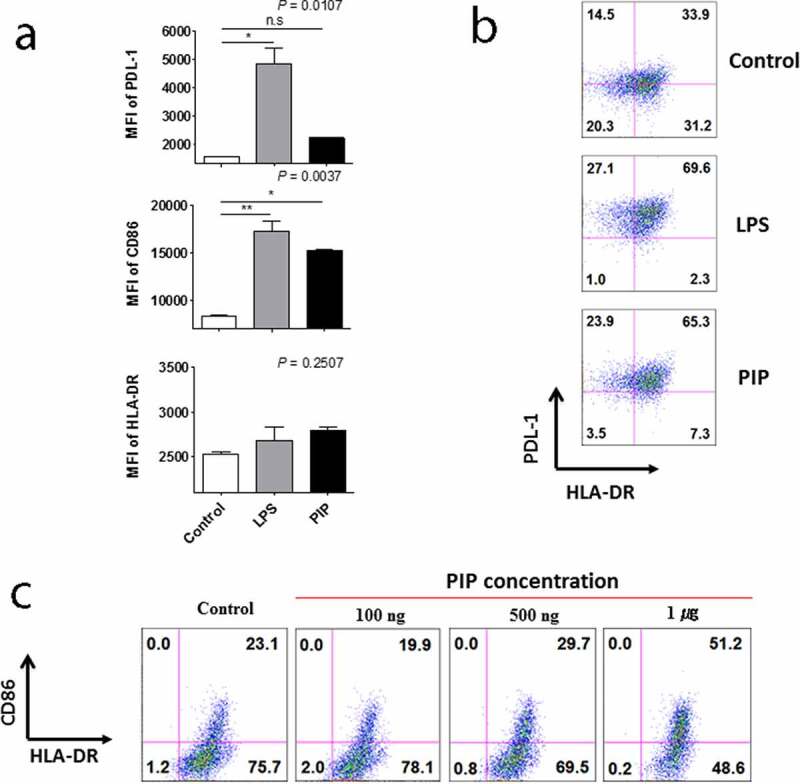
Effect of prolactin-induced protein (PIP) on DC maturation. (a). Expression of HLA-DR, CD86, and PDL-1 on the surface of DCs matured by 1 µg each of LPS and PIP (n = 3). (b). FACS profile of mature DCs. (c). Dose-dependent dendritic cell maturation effect of PIP. The maturation of immature DCs was assessed by FACS analysis of the expression levels of PDL-1, CD86, and HLA-DR on DC surfaces. *p < 0.05; **p < 0.001. Results are representative of three independent experiments. Error bars indicate means ± SD

We attempted to verify proteins that were expressed in CD4^+^ iNKT cells by western blot. Among these proteins, PIP showed a significant effect on DC maturation, leading us to further focus on the characteristics of PIP. We confirmed the mRNA expression pattern of *PIP* in CD4^+^ iNKT cells (Figure 3(a)). Further, PIP protein expression was detected in cells, and its presence was confirmed in the supernatant from CD4^+^ iNKT cell culture by western blotting (Figure 3(b)). In the supernatant, the level of PIP expression increased with the concentration of anti-CD3 Ab used to treat cells (Figure 3(c,d)). We purchased recombinant PIP (OriGene), expressed in HEK293 cells, for further investigation. PIP showed effects similar to that of LPS (Figure 4). Although HLA-DR and PDL-1 were not upregulated, CD86 was significantly upregulated at level similar to LPS treatment (Figure 4(a)), and PIP and LPS induced DC maturation at a rate two times faster than that in PBS (Figure 4(b)). Furthermore, the DC maturation effect of PIP was concentration-dependent (Figure 4(c)).

### Cytokine production from DCs matured by PIP treatment

**Figure 5.**
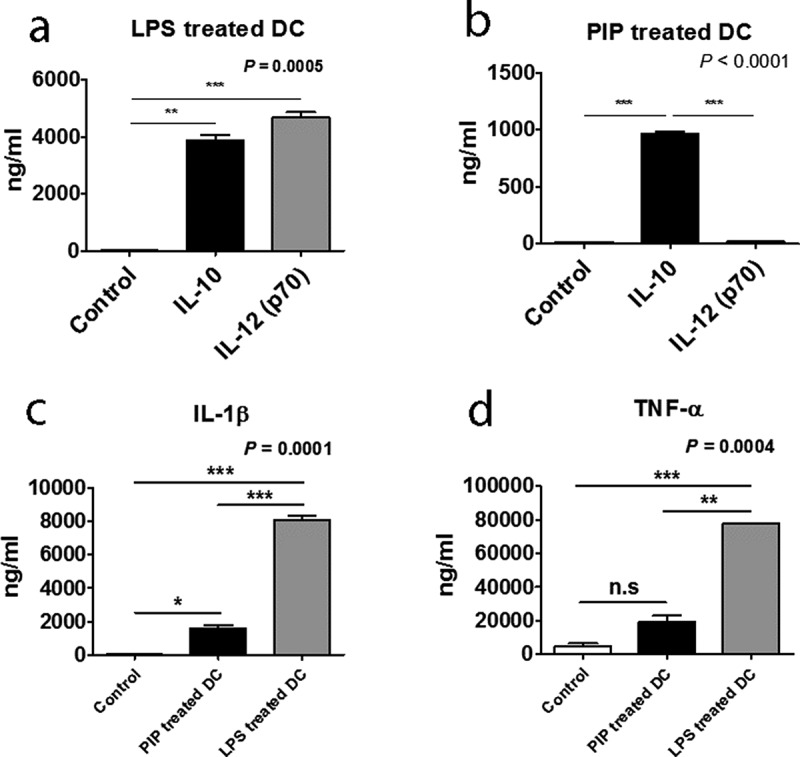
Comparison of IL-10 and IL-12(p70) production in response to LPS, (a), and PIP treatment of DCs, (b). IL-1β, (c), and TNF-α, (d), production by LPS- and PIP-treated DCs. Immature DCs were cultured for 48 hours with PIP or LPS to allow the induction of tolerogenic DCs. Cytokines were analyzed by the Bio-Plex Pro Human Cytokine 17-plex Assay kit. * *p* < 0.05; ** *p* < 0.001; *** *p* < 0.0001. Results are from biological replicate experiments. Error bars indicate means ± SD

We then analyzed the supernatants of DCs matured by LPS and PIP to assess cytokine production. GM-CSF, IL-4, IL-5, IL-7, IL-8, and IL-13 were not detected in the supernatants of immature DCs stimulated by either LPS or PIP. IL-10 production increased compared with that of IL-12(70) following treatment with PIP (n = 2, Figure 5(b)) but not with LPS (n = 2, Figure 5(a)). IL-1β was produced in response to both treatments, but less in response to PIP compared to LPS (n = 2, Figure 5(c)). TNF-α production was not increased significantly in response to PIP, but significantly increased following LPS treatment (n = 2, Figure 5(d)). The other cytokines, G-CSF, IL-17, IL-2, IL-6, IFN-γ, MCP-1, and MIP-1β, were induced to a lesser extent by PIP than LPS (Supplementary Fig. 1).

Mature DCs that were exposed to iNKT cells produced significantly higher levels of IL-10 than IL-12, a phenotype that is consistent with tolerogenic (immunosuppressive properties) function (Figure 5(a,b)) [37].

Among the supernatants of anti-CD3 Ab-stimulated CD4^+^ iNKT, DN iNKT, and conventional CD4^+^ T cells, the CD4^+^ iNKT cells produced the most T_reg_ cells by generating tolerogenic DCs (Supplementary Fig. 2). Therefore, we aimed to identify the factors produced by CD4^+^ iNKT cells that could induce tolerogenic DCs that allowed T_reg_ cells to be generated. Before initiating this study, we also investigated the role of chemokines and cytokines secreted by CD4^+^ iNKT cells in DC maturation, but did not detect any specific cytokines and chemokines involved in T_reg_ generation (Supplementary Table 2).

### PIP generates T_reg_ cells from naïve T cells through Toll-like receptor 2 (TLR2)

**Figure 6.**
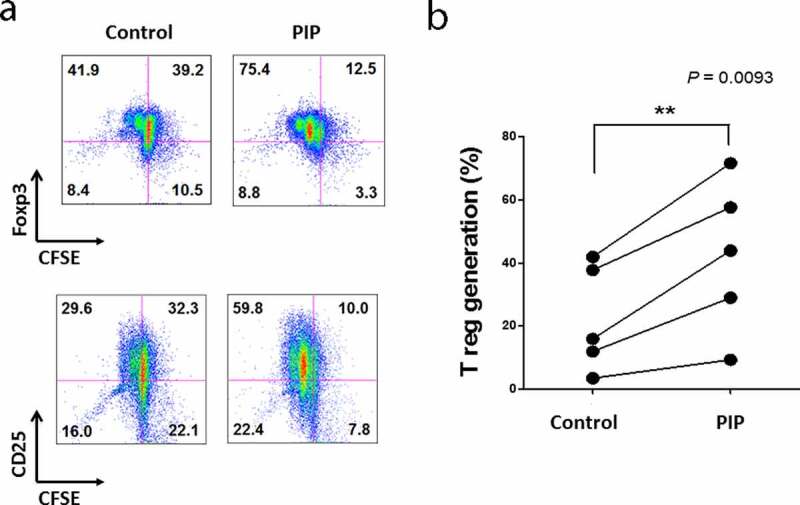
Recombinant prolactin-induced protein (PIP) induces CD4^+^CD25^+^Foxp3^+^ T (T_reg_) cells by inducing the maturation of immature DC. (a). To mature these DCs, CD14^+^ monocytes were cultured with GM-CSF and IL-4 for 5 days. Immature DCs were cultured for 48 h with PIP to allow the induction of tolerogenic DCs. Tolerogenic DCs were co-cultured with CFSE-labeled CD4^+^CD25^−^Foxp3^−^ T cells and 500 ng/ml anti-CD3 Ab, 500 ng/ml anti-CD28 Ab, and 50 U/ml IL-2 for 5 days. The development of T_reg_ cells was assessed by FACS analysis of the expression levels of CD4, CD25, and Foxp3. (b). T_reg_ generation by PIP-treated DCs isolated from healthy volunteers (n = 5)

We investigated the ability of PIP to promote T_reg_ generation, with cells expressing Foxp3 and CD25, and active proliferation phenotype. There was only a 6.8% difference in Foxp3 expression in T cells between the PIP-treated DC and untreated DC groups, whereas PIP-treated DCs caused more active T_reg_ proliferation (PIP, 75.4%) compared to untreated DCs (control, 41.9%). We observed a similar pattern of CD25 expression in T cells, with only a 7.9% difference between the PIP-treated and untreated DC groups; however, in the PIP-treated group, T_reg_ cells more actively proliferated (PIP, 59.8%) than in the untreated group (control, 29.6%) (Figure 6(a) and Fig. S3). In independent experiments performed using a different volunteer’s DCs, we again observed that DCs with PIP treatment induced more active proliferation of T_reg_ cells than DCs without any treatment (n = 5, Figure 6(b)).

**Figure 7.**
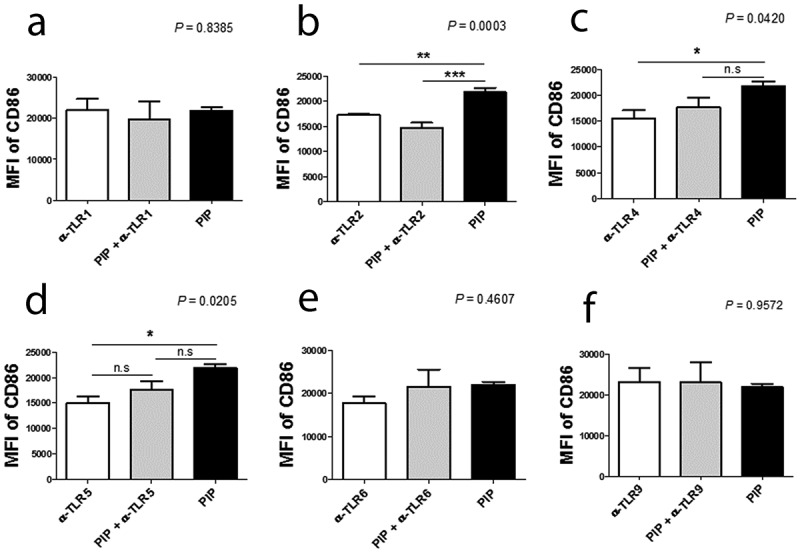
Blocking effect of anti-TLR antibodies on DC maturation. Anti-TLR antibodies (1 µg/ml) were pre-cultured with immature DCs for 1 h before adding 500 ng/ml recombinant PIP. The maturation of immature DC was assessed by FACS analysis of the expression level of CD86 on DC surfaces. * *p* < 0.05. ** *p* < 0.01, *** *p* < 0.001. Results are from biological replicate experiments. Error bars indicate means ± SD

**Figure 8.**
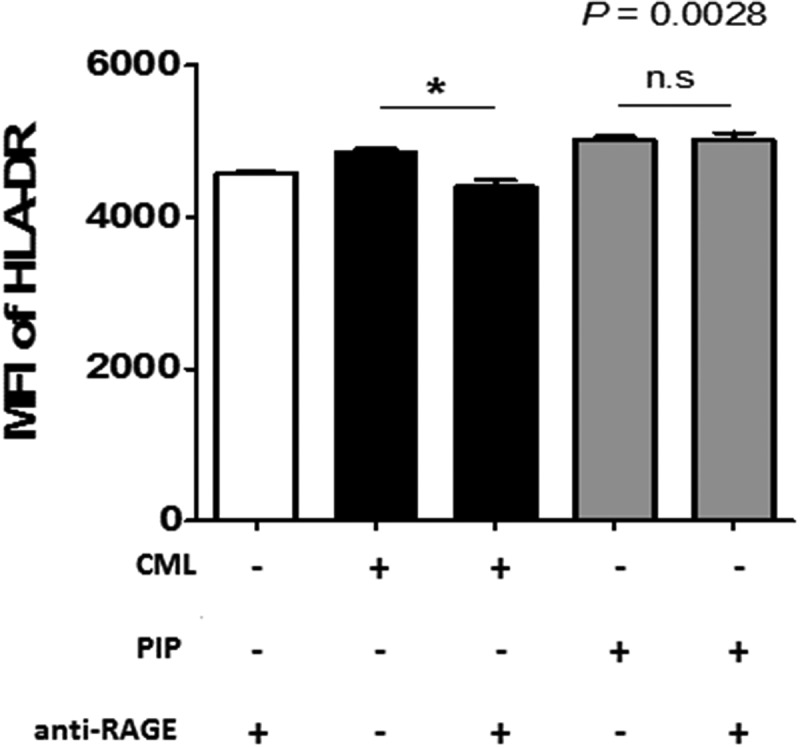
Blocking effect of anti-RAGE Ab on DC maturation. Anti-RAGE Ab (1 µg/ml) was pre-cultured with immature DCs for 1 h before adding 500 ng/ml recombinant PIP. The maturation of immature DCs was assessed by FACS analysis of HLA-DR expression on DC surfaces. *p < 0.05. Results are from biological replicate experiments. Error bars indicate means ± SD

**Figure 9.**
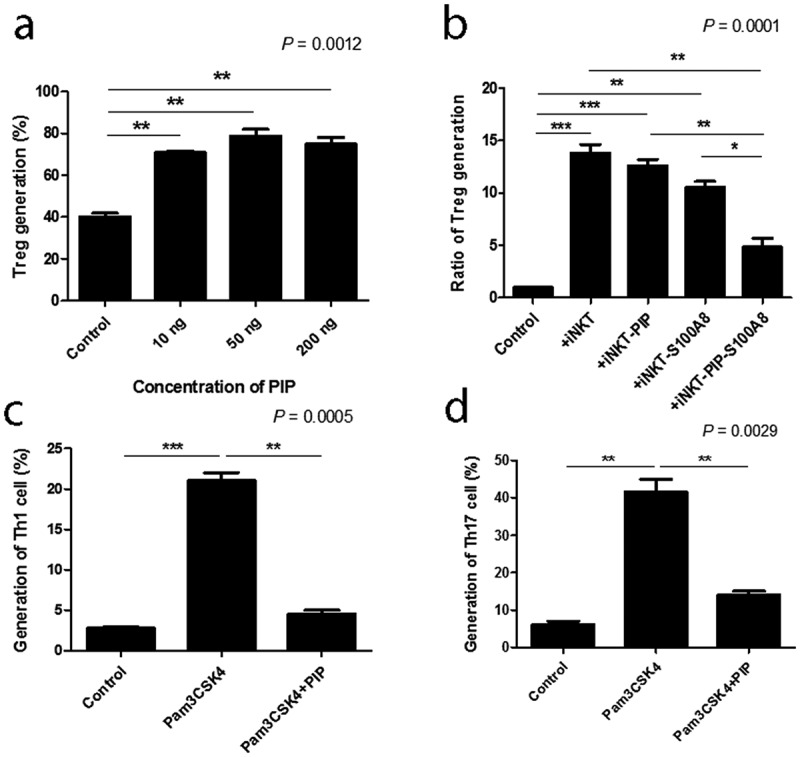
Generation of T_reg_ cells with various concentrations of recombinant PIP and blocking the generation of Th1 and Th17 cells. (a). Tolerogenic DCs were induced by treatment with PIP. CFSE-labeled CD4^+^CD25^−^Foxp3^−^ T cells were added to tolerogenic DCs to evaluate T_reg_ generation by FACS analysis. (b). Inhibition of T_reg_ generation by PIP siRNA and/or S100A8 siRNA treatment of CD4^+^ iNKT cells. (c). Inhibition of Th1 cells by Pam3CSK4 through T_reg_ cells generated by recombinant PIP. (d). Inhibition of Th17 cells by Pam3CSK4 through T_reg_ cells generated by recombinant PIP. Immature DCs were matured with PIP and/or Pam3CSK4 together. CFSE-labeled naïve T cells were added to mature DCs to evaluate Th1 and Th17 cell generation by FACS analysis after 8 days. Results are from biological replicate experiments. Error bars indicate means ± SD

DC maturation process can be induced indirectly through contact with cytokines released by local nonimmune or immune cells that have been stimulated via their own TLRs, or by direct stimulation mediated through DC-expressed TLRs. iNKT cells can trigger enhanced T cell responses once activated by a prevailing stimulus, such as glycolipid α-GalCer, implying that iNKT cells can deliver all essential signals that are vital for DC activation [38]. In the ontogeny of autoimmunity, the exact mechanism controlling the generation of pro-inflammatory DCs, opposite to tolerogenic DCs, remains a crucial question. Interestingly, DC maturation by PIP was blocked by adding anti-TLR2 Ab (n = 2, Figure 7(b)). Further, *N^Є^*–carboxymethyllysine (CML), an agonist of the receptor for advanced glycation endproducts (RAGE) [39], did not induce DC maturation, and anti-RAGE Ab did not inhibit the maturation effect of PIP (n = 2, Figure 8). This suggests that PIP signaling for DC maturation might be mediated by TLR2 and not RAGE. Even small amounts of PIP (10 ng) induced T_reg_ cells (n = 2, Figure 9(a)). When PIP siRNA or S100A8 siRNA was added individually into the DC maturation mixture from CD4^+^ iNKT cells, no significant reduction in T_reg_ cell generation was observed. In contrast, PIP siRNA, together with S100A8 siRNA reduced T_reg_ cell generation significantly (n = 2, Figure 9(b)). The generation of T_reg_ cells by CD4^+^ iNKT cells is interesting as it suggests the presence of potent immunoregulatory functions of iNKT cells in the development of inflammatory diseases. Therefore, it appears reasonable to hypothesize that CD4^+^ iNKT cells effectively regulate the differentiation of pro-inflammatory Th1 and Th17 cells by generating T_reg_ cells [40,41]. PIP and Pam3CysSerLys4 (Pam3CSK4), a synthetic bacterial lipoprotein and TLR2 and TLR1 ligand, used to treat DCs with CD4^+^CD25^−^Foxp3^−^ T cells, showed a substantial suppressive action on the differentiation of Th1 and Th17 cells (n = 2, Figure 9(c,d)). These data indicate that CD4^+^ iNKT cells induce T_reg_ cells, which subsequently suppress Th1 and Th17 development even under pro-inflammatory conditions. It has been suggested that Foxp3 transcriptionally suppresses Th1 and Th17 cell differentiation [38]. However, the data provided here suggest the possibility that T_reg_ cells suppress the differentiation of pro-inflammatory Th1 and Th17 cells.

## Conclusion

**Figure 10.**
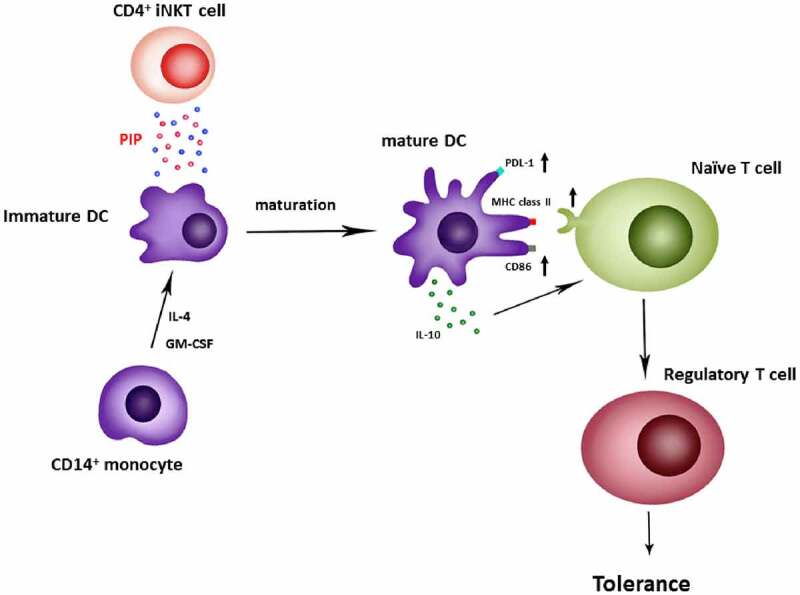
Generation of regulatory T cells through PIP secreted from CD4^+^ iNKT cells. Anti-CD3/anti-CD28 Ab-activated CD4^+^ iNKT cells secrete PIP to upregulate PDL-1, MHC class II, and CD86 expression on the surfaces of DCs. Matured DCs also secrete IL-10 cytokine. Finally, mature DCs induce regulatory T cell proliferation from naïve T cells

In the present study, we identified a new function of PIP, that is T_reg_ generation through the induction of tolerogenic DCs. Peripheral immune tolerance is closely related to sustained tumor growth. Therefore, blocking PIP expression in tumor cells may be the first step toward preventing the immune escape of cancer cells, with a subsequent increase in cytotoxic T cells that enable the clearance of tumor cells[42]. In addition, in the mucosal environment, PIP may produce T_reg_ cells in response to allergens to promote anergy, tolerance, and active suppression [43,44]. Taken together, we report here that PIP, a key factor secreted by CD4^+^ iNKT cells, promotes tolerogenic DC maturation [45] involved in T_reg_ generation (Figure 10).

## Supplementary Material

Supplemental Material

## Highlights

- iNKT cells dysfunction in cytokine secretion is associated with autoimmune disease.
- PIP induces the differentiation of naïve T cells into CD4^+^CD25^+^Foxp3^+^ T_reg_ cells.
- PIP reduces the number of Th1 and Th17 cells produced by Pam3CSK4.
- PIP mediates immunoregulation by iNKT cells through TLR2-mediated sigaling.
