# Supplementary material for: Peripheral immune tolerance by prolactin-induced protein originated from human invariant natural killer T cells
Source: Bioengineered. 2021 Jan 28;12(1):461–75. doi: 10.1080/21655979.2021.1875664 (PMC8806214; doi:10.1080/21655979.2021.1875664)
Supplement: Supplemental Material [file KBIE_A_1875664_SM4112.zip › supplement/Supplementary Figure.docx]

**Supplementary Figure 1. Cytokine production in response to LPS (A), and PIP (B).** Production of pro-inflammatory cytokines by PIP-treated immature DCs is reduced. Immature DCs were cultured for 48 h with PIP to allow the induction of tolerogenic DCs. Cytokines were analyzed by the Bio-Plex Pro Human Cytokine 17-plex Assay kit (Bio-Rad). * *p* < 0.05; ** *p* < 0.001; *** *p* < 0.0001. Results are from biological replicate experiments. Error bars indicate means ± SD

**Supplementary Figure 2. DC maturation and regulatory T cell generation in response to CD4^+^ T cell, CD4^+^ iNKT cell, or DN iNKT cells. A**. Human immature DCs differentiated into mature DCs with the addition of supernatant from anti-CD3 Ab-activated CD4^+^ T cells, CD4^+^ iNKT cells, and DN iNKT cells. The data were obtained from duplicate experiments with each clone. **B**. CD4^+^ iNKT cells preferentially enhanced Foxp3 expression compared to CD4^+^ T cell and DN iNKT cells. The data presented are representative of three experiments. Differences were evaluated by one-way ANOVA, followed by Bonferroni’s multiple comparison tests. ***p* < 0.001. Error bars indicate the means ± SD (Cited from Ref 28).

**Supplementary Figure 3.** **Regulatory T cell generation in response to recombinant PIP-induced DC maturation.** Human immature DCs differentiated into mature DCs following the addition of recombinant PIP. **A.** Gating strategy of T cells. **B.** Recombinant PIP-treated DCs preferentially enhanced Foxp3 expression compared to non-treated DCs. The data presented are representative of three experiments.
