# Supplementary material for: Peripheral immune tolerance by prolactin-induced protein originated from human invariant natural killer T cells
Source: Bioengineered. 2021 Jan 28;12(1):461–75. doi: 10.1080/21655979.2021.1875664 (PMC8806214; doi:10.1080/21655979.2021.1875664)
Supplement: Supplemental Material [file KBIE_A_1875664_SM4112.zip › supplement/Supplementary_Tables_Lee_et_alclean.docx]

**Supplemantary Table 1** The supernatant of CD4^+^ iNKT cells’ protein components analyzed by Mass spectrometry

* Group A; The protein which was detected 8 times of 9 sample analysis of 4 independent experiments. Group B; The protein which was 7 times. Group C; The proteins which were detected 6 times. Group D; The proteins which were detected 5 times. (Cited from Ref. 27)


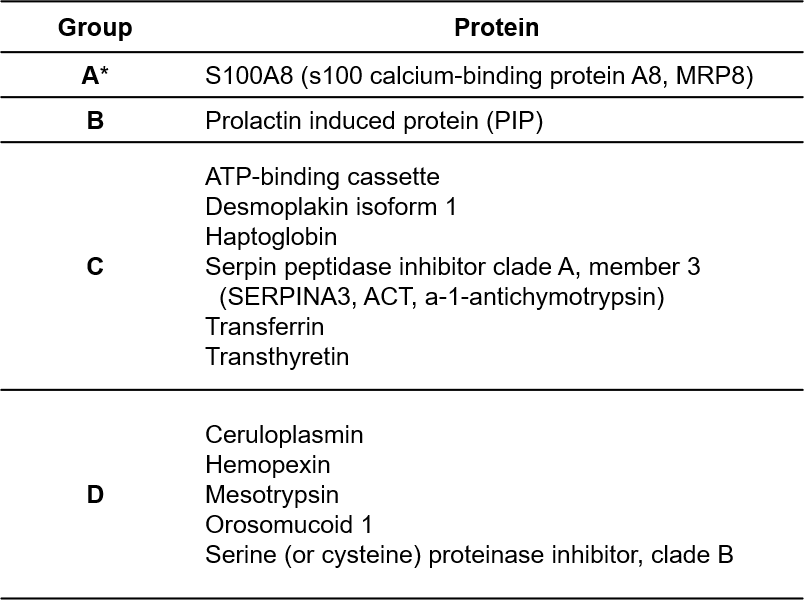


**Supplemantary Table 2** Cytokine and chemokine profiling of supernatant of CD4^+^ and DN iNKT cells


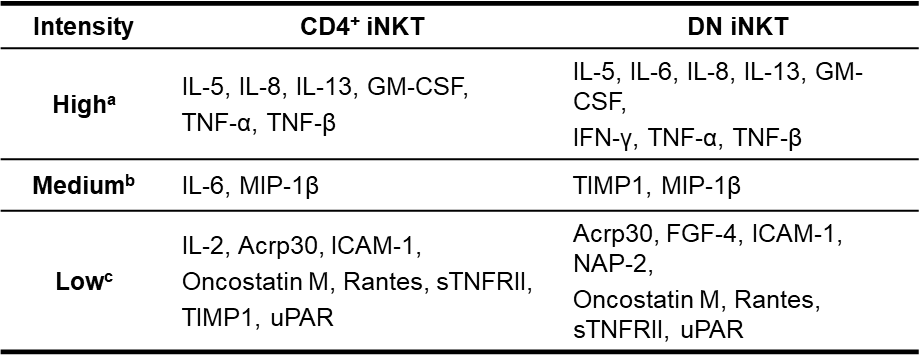


^a^Cytokines which were detected within a minute in ELISA reaction. ^b^Cytokines which were detected between 1 and 5 minutes in ELISA reaction. ^c^Cytokines which were detected between 5 and 10 minutes in ELISA reaction. (Cited from Ref. 27)
