# Supplementary figures and images for: Peripheral immune tolerance by prolactin-induced protein originated from human invariant natural killer T cells
Source: Bioengineered. 2021 Jan 28;12(1):461–75. doi: 10.1080/21655979.2021.1875664 (PMC8806214; doi:10.1080/21655979.2021.1875664)

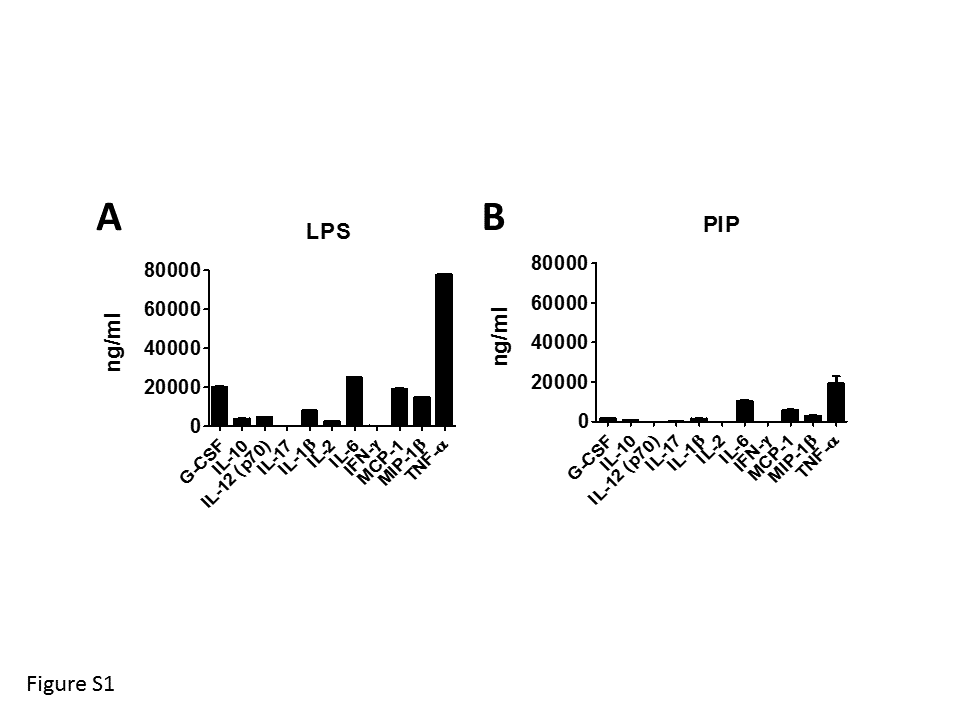

Supplement: Supplemental Material [file KBIE_A_1875664_SM4112.zip › supplement/Fig_S1.png]

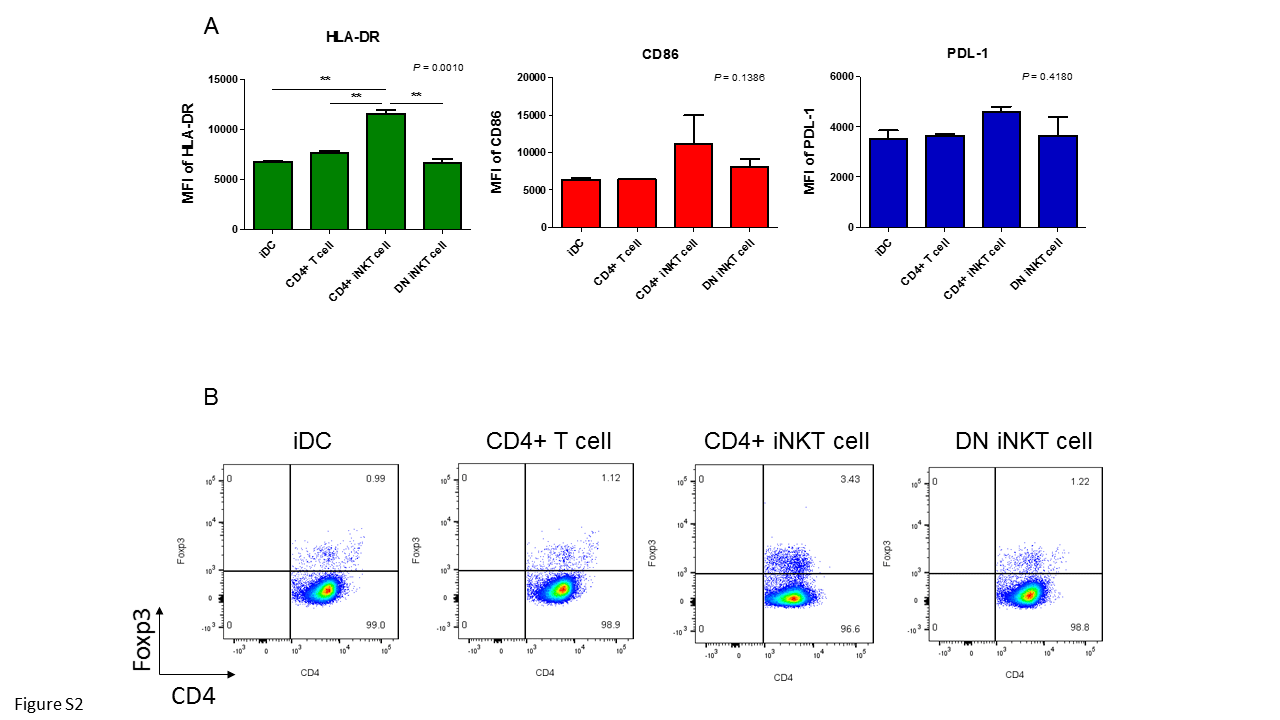

Supplement: Supplemental Material [file KBIE_A_1875664_SM4112.zip › supplement/Fig_S2.png]

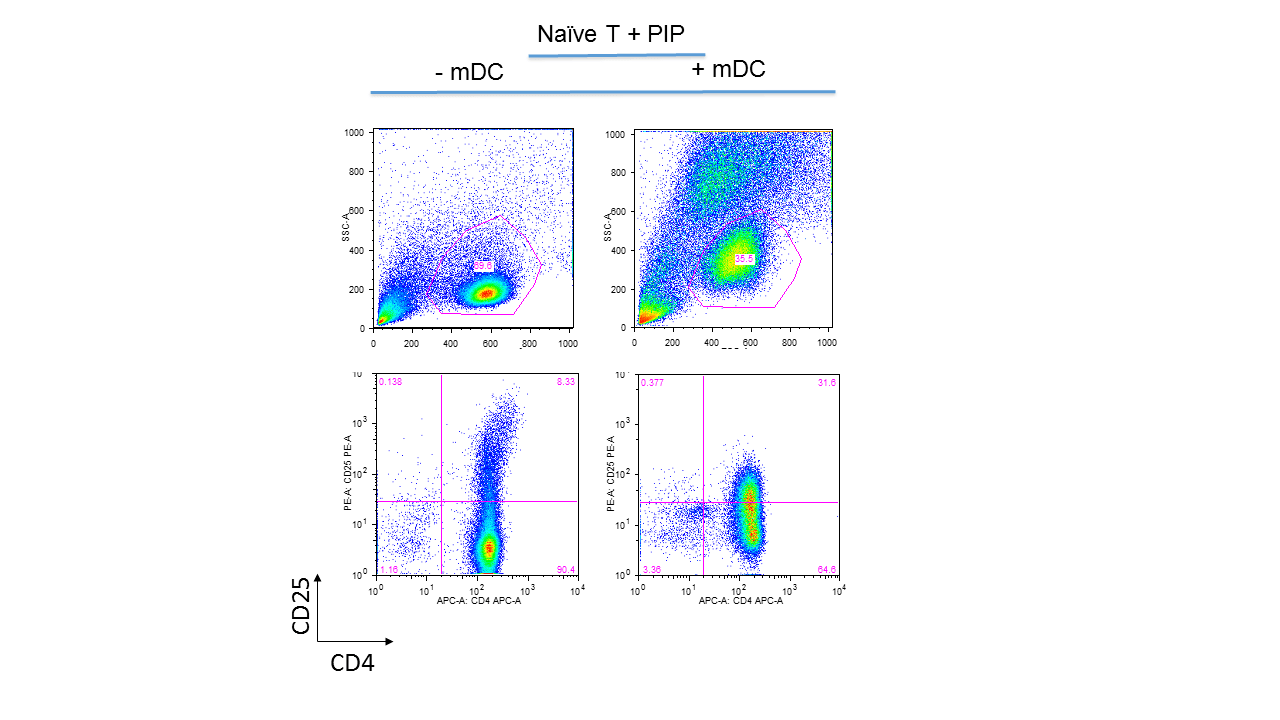

Supplement: Supplemental Material [file KBIE_A_1875664_SM4112.zip › supplement/Fig_S3.png]
